# Metabolic Responses of Bacterial Cells to Immobilization

**DOI:** 10.3390/molecules21070958

**Published:** 2016-07-22

**Authors:** Joanna Żur, Danuta Wojcieszyńska, Urszula Guzik

**Affiliations:** Department of Biochemistry, Faculty of Biology and Environmental Protection, University of Silesia in Katowice, Jagiellonska 28, 40-032 Katowice, Poland; jozur@us.edu.pl (J.Ż.); danuta.wojcieszynska@us.edu.pl (D.W.)

**Keywords:** immobilization, immobilized cells, metabolic response, biofilm

## Abstract

In recent years immobilized cells have commonly been used for various biotechnological applications, e.g., antibiotic production, soil bioremediation, biodegradation and biotransformation of xenobiotics in wastewater treatment plants. Although the literature data on the physiological changes and behaviour of cells in the immobilized state remain fragmentary, it is well documented that in natural settings microorganisms are mainly found in association with surfaces, which results in biofilm formation. Biofilms are characterized by genetic and physiological heterogeneity and the occurrence of altered microenvironments within the matrix. Microbial cells in communities display a variety of metabolic differences as compared to their free-living counterparts. Immobilization of bacteria can occur either as a natural phenomenon or as an artificial process. The majority of changes observed in immobilized cells result from protection provided by the supports. Knowledge about the main physiological responses occurring in immobilized cells may contribute to improving the efficiency of immobilization techniques. This paper reviews the main metabolic changes exhibited by immobilized bacterial cells, including growth rate, biodegradation capabilities, biocatalytic efficiency and plasmid stability.

## 1. Introduction

For many decades attention has been focused on microbial behaviour in planktonic systems, although it has been reported that in natural environments, and clinical and industrial settings a wide range of surfaces constitute the major sites of microbial occurrence. Bacteria may grow planktonically or form a biofilm, a multicellular structure, which adheres to a surface and is stabilized by a self-produced matrix. In natural communities of bacteria it has been documented that attached microorganisms are more active than their free-living counterparts, and exhibit differences in gene expression [1,2]. Furthermore, it is well known that interaction between bacteria and solid phase results in a variety of physiological changes in microbial behaviour [3,4,5,6,7,8]. As long ago as 1943 ZoBell [9] demonstrated that bacterial activity increased due to the presence of a glass support, even when nutrient concentrations in the environment were low [4,9]. In recent years immobilized cell (IC) systems have been commonly used for biotechnological purposes, e.g., in bioremediation and biodegradation, biocontrol, pesticide application, and the production of various compounds, such as amino acids, antibiotics, steroids or enzymes. However, data about the effects exerted by immobilization on microbial physiology remain limited and widely dispersed [10,11,12,13]. Moreover, immobilization of living and growing cells due to their self-proliferating and self-regenerating properties, and their ability to catalyze multistep and multifunctional reactions involving coenzyme regeneration may be used for various purposes.

## 2. Conditions of Bacterial Cells Immobilization

Cell immobilization is a general term describing the physical confinement of viable microbial cells to a certain defined region of space (carrier) in order to limit free migration and exhibit hydrodynamic characteristics different from those of the surrounding environment [14,15]. Compared with systems utilizing suspended microorganisms, immobilized cells technology offers numerous advantages, e.g., continuous utilization, higher cell density, higher metabolic activity, retention of plasmid-bearing cells, prevention of interfacial inactivation, better productivity, protection against acidification and shear forces in the environment, and resistance to heavy metals, solvents, pH and temperature [1,13,15,16]. Immobilized cell systems are far more tolerant to changing environmental conditions and less vulnerable to toxic substances present in the bulk phase. General techniques used for immobilization include: flocculation, adsorption on surfaces, covalent bonding to carriers, cross-linking of cells, entrapment, encapsulation and nanocoating [10,14,17]. It must be noted that the great majority of studies on the use of viable immobilized microbial cells have been performed at the laboratory scale. Limitations on the application of IC systems on an industrial scale are mainly attributed to mass transfer limitations within the supports, and coupled additional transfer processes. Factors affecting the efficiency of the immobilization (adsorption) of microbial cells depend on the properties of the support, microbial cell surface and environmental conditions [10,11,14,18]. Factors determining the adsorption of microbial cells are listed in Table 1.

**Figure 1:**
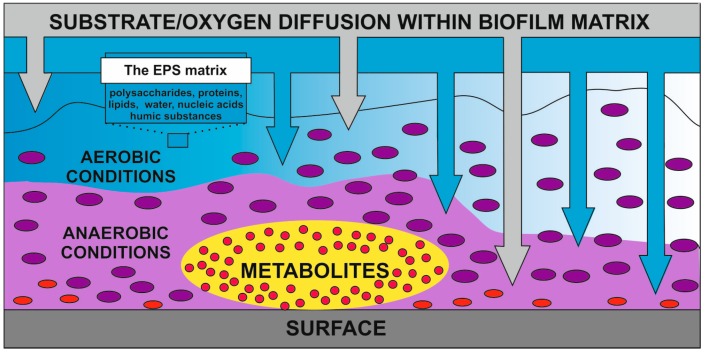
Substrate and oxygenic heterogeneity of biofilm [1,6,16,22,23].

One of the most frequently used techniques for whole cell immobilization is adsorption on a surface. Factors which may affect the metabolic activity of bacterial cells on surfaces include: changes in pH, the concentration of substrates and ions, the presence and concentration of inhibitors, and the release of metabolites from cells [14,19,20]. The adhesion of microorganisms is frequently increased in the exponential growth phase due to increased cell wall hydrophobicity, and thereby surfaces are the preferred locus for metabolically active bacteria [4,21]. The most significant changes in microbial metabolism observed in cells immobilized by adsorption are mainly due to biofilm formation. Biofilm is a complex surface-attached or associated with the interfaces of microbial communities formed in response to specific environmental conditions, such as nutrient and oxygen availability (Figure 1) [16,22,23].

**Figure 2:**
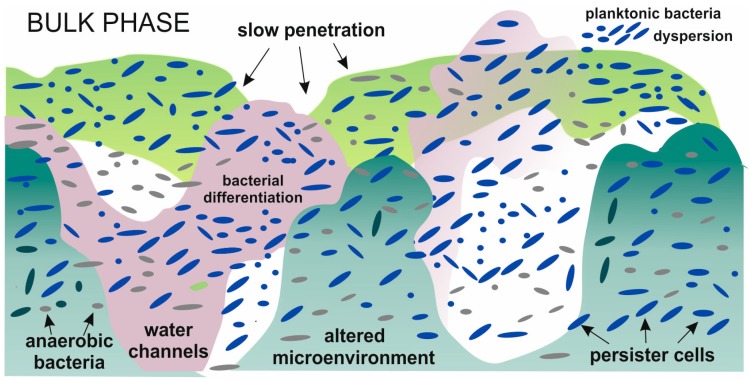
Physiological and structural heterogeneity of biofilm [1,6,22].

Stewart et al. [24] point out that in biofilms two self-assembly phenomena can be distinguished: molecular self-assembly and colloidal self-assembly. The first of them describes the associations between matrix components; the second refers to the formation of the biofilm itself. In the second phenomena cells combine with the polysaccharides and proteins of the extracellular polymeric substances (EPS) to produce a viscous and elastic material. These colloidal interactions, which are formed due to physical interactions between suspended cells and polymeric structures, are responsible for biofilm morphology and mechanics. It is known that microorganisms undergo diverse and profound changes during their transition from suspended in solution to sessile communities [6,25]. Cells in biofilms are characterized by structural and physiological heterogeneity due to the formation of spatial scales and altered microenvironments within the layers (Figure 2).

Most bacterial species are capable of biofilm formation, and its development is generally considered as a universal strategy for bacterial survival, since biofilms protect microorganisms from variable environmental conditions [2,7,11,16,26]. During biofilm development bacteria produce high molecular weight biopolymers enabling cell-to-cell and cell-surface/interface attachment called, as mentioned above, extracellular polymeric substances (EPS, exopolysaccharide, exopolymer, microbial flocculants, biopolymers). EPS is mainly composed of polysaccharides, proteins, lipids, extracellular DNA (eDNA), surfactants and humic substances, and it is a major component in microbial aggregates responsible for keeping cells together in a three-dimensional structure [27]. Proteomic analyses conducted by Junter and Jouenne [12] showed changes in bacterial protein profiles between suspended and immobilized cells ranging from 3% to even more than 50% of the examined proteins. Changes included three major groups: proteins involved in the early step of biofilm formation and attachment of bacteria, proteins responsible for cofactors and amino acid biosynthesis, and proteins involved in the adaption and protection of cells. Proteomic differences are observed not only between immobilized and free-floating bacteria, but even among different types of biofilms, e.g., floccular and granular [28]. Several types of EPS can be distinguished: capsular (C-EPS), slime (S-EPS), loosely bound (LB-EPS) and tightly bound (TB-EPS). More et al [29] reported that organic compounds secreted by microorganisms can be divided into three major groups. First type is produced by bacteria under the influence of interaction with the environment, second type is secreted due to the substrate metabolism, and the third type is associated with bacterial growth and metabolites released during cell lysis and/or biodegradation of microbial components [30]. The important factors affecting EPS production include genotype, growth phase, carbon and nitrogen ratio and their sources, level of phosphorus, micronutrients, trace elements, vitamins, metals, pH, temperature, aerobic or anaerobic conditions, and pure or mixed culture. EPS mediates in mass transfer through biofilm, adsorption of xenobiotics, metals or inorganic ions, and provides physical support for the formation of biofilm (Table 2) [2,29,31,32,33].

## 3. Metabolic Responses to Immobilization

Evaluation of the influence of immobilization on bacterial physiology is difficult to define, mainly due to the taxonomic, genetic and functional differences between bacteria. Divergences in the experimental data are partly due to the great variation in the applied carriers, species of bacteria, techniques of immobilization or culture conditions. Detailed reviews about microbial activities at interfaces have been published [4,11,38], while the influence of immobilization on microbial activity remains poorly understood. Moreover, there are some controversies regarding the influences of immobilization upon bacterial activity [12,39]. Van Loosdrecht et al. [4] also noticed that most authors have not distinguished between the direct and indirect effects of surfaces upon bacterial activity. Despite this, there are several major activities in which the assessment of changes and general observations may be made (Table 3) [8,11].

### 3.1. Growth Rate

In studies concerning the effects of fixation on microbial growth rate a lot of contradictory results have been published. Nevertheless each of them, namely increased [47,48,49,50,51], decreased [40,41,42] or unchanged growth [8,52] have an explanation (Table 4).

As Cassidy et al. [10] and Smet et al. [8] noticed, in general, the nature of effects depends on bacterial species, type of carrier, initial inoculum size, and the culture conditions that have been used. It is noteworthy that cells attached in natural settings exhibit significant growth, whereas artificially immobilized cells are allowed to limit [8,13]. Conditions that promote growth of attached bacteria have been attributed to the tendency for dissolved nutrients and particles to be adsorbed on the surface, thus making them more available for bacteria [63,64]. This mainly concerns high molecular weight compounds with multiple free functional groups which serve as binding sites. A lot of macromolecules assimilated by bacteria must firstly be hydrolyzed by bacterial enzymes [65]. Since the dissolved solutes are adsorbed on surfaces, the immobilization may promote access of enzymes to the adsorbed nutrients. On the other hand, adsorption of macromolecular substances may have an adverse effect. Surface coating by a layer of adsorbed substrates could prevent or decrease their hydrolysis [10,11]. Ellwood et al. [47] suggested that increasing the growth rate of surface-associated bacteria was connected with an increase in local proton concentration between the bacterial cell and the solid surface. This results in an enhancement of the efficiency of proton re-uptake. In these conditions more energy may be available to the bacterial cell and may encourage growth [47]. This effect was also observed for *Escherichia coli* K-12, which exhibited enhanced metabolic activity after adhesion on a glass surface [66]. Keen and Prosser [48] suggested that the increased growth rate of *Nitrobacter* cells attached to glass, and a broader range of pH in which bacteria were able to grow are due to the extracellular slime layer formed by surface-associated cells. The slime layer decreased nitrite concentration, which in high concentrations is toxic to them. A positive effect of the presence of the slime layer was also observed for *Pseudomonas aeruginosa*, capable of mercury adsorption [67]. Jobby et al. [68] noticed also that the bacterial cell wall is negatively charged due to the presence of anionic structures, which allowed cation binding, hence, the slime layer or capsules found in some bacteria are significant structures in, e.g., heavy metal binding [68]. It is likewise noteworthy that large amounts of bacteria associated with the surface may counteract losses in biomass resulting from variable dilution rates.

The second explanation for the increasing growth rate observed after immobilization is the protection provided by the supports [11,12,13]. Support selection is one of the essential criteria for successful immobilization [15]. There are two types of carriers: inorganic materials and organic polymers. Regardless of type, a suitable carrier should be non-toxic and non-polluting, light weight, have a high mechanical and chemical stability, high diffusivity, and biomass retention, minimal attachment of other organisms, and preferably inexpensive [14,17]. 

**Figure 3:**
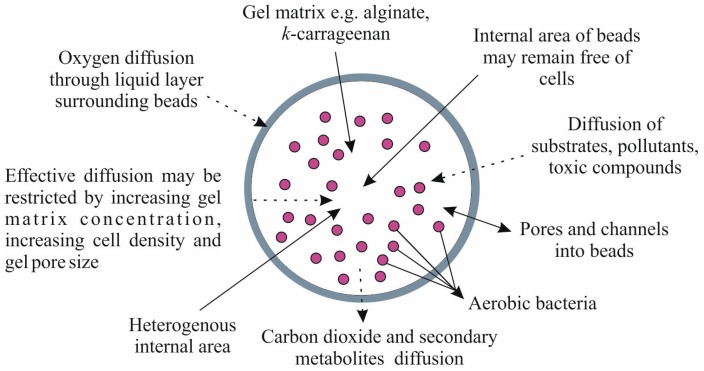
Schematic diffusion in gel beads [10,69].

The first of the most reliable reasons for the decreased growth rate observed in immobilized cells is the formation of the oxygen and nutrient gradients within the supports, as a consequence of mass transfer limitation [8,11,42,69]. Decreased growth rate is also attributed to product inhibition arising from the mass transfer-limited removal of acidic secondary metabolites. Immobilized cells focus on the periphery of the carriers, while the inner parts without nutrients remain free. The accumulation of bacteria at the edge of capsules may result in the weakening of beads and the release of bacteria into the medium. Most studies about the diffusion of substrates and oxygen in immobilized cells applied alginate and k-carrageenan beads (Figure 3).

Cassidy et al. [10] and Zhao et al. [69] noticed that, for example in soil, the thin liquid film surrounding the beads may play a crucial role in the limitation of gas diffusion, especially oxygen. Limitations in gas diffusion particularly concern the internal area of carriers, which is often not homogeneous due to various factors (temperature, type of carrier, porosity, viscosity). Results obtained by Meldrum et al. [41] confirmed that the decreased growth rate of *Listeria monocytogenes* Scott A presumably resulted from potential stresses and diffusion limitation associated with physiological differences between the planktonic and immobilized state of cells. Goodman and Marshall [70] also noticed that restricted gas diffusion and development of the pH gradient can alter the expression of certain genes. 

Different values of the diffusion coefficient in beads for many substances have been reported. Determining the optimal diffusion coefficient of substrates plays a crucial role in maintaining the kinetic behaviour of immobilized cells in the supports [71,72]. Golmohamadi and Wilkinson [72] highlight that in Ca-alginate hydrogels ion diffusion is a crucial factor which determines solutes penetration. Moreover, according to obtained data, diffusion depends mainly on the physicochemical structure of the hydrogel. Tanaka et al. [71] investigated the diffusion characteristics for several substrates, such as glucose, l-tryptophan and α-lactoalbumin. As the results showed, the diffusion of the examined substrates was freely into and from the gel beads without disturbance by the pores in the beads [71]. Oxygen supply to bacteria entrapped in alginate also depends on the physiological status of microorganisms [73]. However, in most cases decreased substrate and oxygen diffusion are mainly attributed to the increased cell loading within beads, the increasing supports concentration, the negative superficial charge of most carriers, and pore diameter [42]. Many authors also highlight that studies about substrates utilization in biofilms should be calculated with the use of diffusion-reaction model [74,75]. Studies conducted by Beyenal and Tanyolaç [74] demonstrated that the effective diffusion coefficients of glucose, ammonium ion and oxygen were dependent on biofilm density of *Zooglea ramigera* immobilized on activated carbon particles. Yu and Pinder [75] on the basis of decrease in diffusivity of lactose in acidogenic biofilms suggested that increased solid biomass fraction of biofilms may influence on efficiency of substrates diffusion. Lack of changes in growth rate between planktonic and immobilized cells has rarely been observed. Nevertheless, van Loosdrecht et al. [4] suggested that it may result from an absence of adsorbed nutrients, although the literature data on this subject are very scarce. However, lack of differences between the growth rate of free and entrapped in alginate beads *Acinetobacter johnsonii* cells was reported by Muyima et al. [52]. Boons et al. [76] investigated the effects of immobilization and salt concentration on the growth dynamics of *Escherichia coli* K-12 and *Salmonella typhimurium*. The obtained data revealed that immobilization in gelatin and xanthan gum only affected the lag phase at high salt concentration for both microorganisms [76]. Zhang et al. [77] used *Shewannela oneidensis* MR-1 as a model organism in order to elucidate main physiological differences occurring between cells embedded in a self-produced matrix and cells that are immobilized in an alginate hydrogel. Obtained results showed no significant differences in growth rate, cell viability, surface charge and hydrophobicity between cells of those two examined systems. The growth of the cells physically entrapped in the alginate hydrogel were characterized by a higher requirement for metabolic energy and lowered siderophore-mediated iron uptake, but the most valuable observation concern that the presence of the alginate hydrogel results in decreased production of proteins involved in biofilm formation and simultaneously induces the higher production of eDNA.

### 3.2. Biocatalytic Efficiency/Changing Yields or New Metabolic Behaviour of Immobilized Cells

Improvement in the productivity of immobilized cells could be achieved by a substantial increase in immobilized biomass density and by using high flow rates in continuous systems. Currently, the production of specific metabolites, e.g., antibiotics, organic acids, amino acids or alcohol, is one of the major applications of immobilized cell systems [13,14,15]. It must be noted that mass transfer limitation occurring in immobilized cell systems are in some cases responsible for unchanged or even decreased productivity as compared to free-living cells. However, the overwhelming majority of studies refer to yeast, although some data about bacteria are also available. Some authors [56,78,79] have suggested that changes in metabolic patterns or increasing metabolic efficiency result from decreased water activity and oxygen supply, two factors affecting the microenvironment of immobilized cells. According to a hypothesis explaining the major reasons responsible for changes, rearrangements in the intracellular pools of metabolites result in the increasing productivity of a certain metabolite. Simultaneously, the balance between coenzymes NADH and NADPH in the immobilized cells is frequently altered. In 1981 Esener et al. [80] observed that low water activity improves the maintenance of metabolism of the immobilized cells, which results in decreased cell growth. Immobilization techniques are suitable not only for wild type bacteria, but they may also be successfully used for mutant strains. Branco et al. [81] used *Ochrobactrum tritici* As5 with inactivated arsenite efflux pumps immobilized in poly (tetrafluoroethylene) for arsenite biofiltration. Two mechanisms of microbial metals biosorption can be distinguished: metabolism-dependent, occurring only in viable microbial cells, and metabolism-independent, due to the presence of functional groups in cell envelopes. Biosorption can also be classified as extracellular and intracellular accumulation, cell surface sorption and precipitation [82]. Zhang et al. [83] observed that immobilization of annamox bacteria on magnetic porous carbon microspheres increased bacterial retention, but primarily reduced biological, organic and inorganic membrane fouling. Ebrahiminezhad et al. [84] showed enhanced menaquinone-7 production (15% higher yield as compared to control) for *Bacillus subtilis natto* immobilized on magnetic nanoparticles. 

A comprehensive study on the addition of ion-exchange resins and their impact on bacterial activity was carried out by Hattori and Hattori [43,85]. The results of this study indicated that the addition of ion-exchange resins decreased the rate of substrate oxidation. Presumably, lower oxidation resulted from a reduction of the surface area of the attached bacteria that was exposed to the liquid medium and thus nutrients and oxygen. The observed inhibition may also result from the removal of cofactors by the resins. The addition of anionic and cationic resins results in an increase or decrease in the pH optimum, respectively. According to Hattori and Hattori [43,85], the slight negative superficial charge due to bound anions on the anion-exchange resin attracts protons. As a consequence, attached cells are exposed to the higher concentration of hydrogen ions compared to their free counterparts in the bulk phase. However, van Loosdrecht et al. [4] highlighted that it is doubtful that protons associated with the surface will have any significant influence on bacterial activity. They suggested that the pH value of the medium itself is changed after the addition of resins due to the exchange of chloride ions with hydroxyl and phosphate ions. The last observations concern the shift from more reduced to more oxidized metabolites of glucose fermentation by *Escherichia coli*. Van Loosdrecht et al. [4] suggested that the selective binding of anionic fermentation products results in an observed shift of dissolved metabolites in liquid medium. 

Another major task in the field of applied microbiology using immobilized cells is antibiotic and chemotherapeutics production [13,86]. Supports used for antibiotics synthesis by IC systems include, e.g., Ca-alginate, polyacrylamide, *k*-carrageenan, cotton or Celite. Antibiotics produced by immobilized bacterial cells include actinomycin D, bacitracin, cephalosporins, chlortetracycline, erythromycin and neomycin [13]. El-Naggar et al. [55] investigated the correlation between support material and antibiotic MSW2000 production by *Streptomyces violatus*. *S. violatus* adsorbed on sponge cubes yielded the highest antibiotic concentration. Simultaneously, entrapment in Ca-alginate beads, even compared to free cells, gave a relatively low antibiotic concentration. Presumably, oxygen diffusion trough alginate beads was insufficient, and thus may affect the metabolic activity of *S. violatus*. As Stormo and Crawford [87] suggested, the porosity of the beads and the size of the diffusing molecules may alter the antibiotic recovery from the medium. Moreover, El-Naggar et al. [55] noticed that cultivation in static cultures increased the production of MSW2000 approximately 3-fold of that obtained in a shaken one. The higher yields of MSW2000 production may be due to the better availability of starch adsorbed to the sponge’s intercellular and extracellular spaces. The sponge-adsorbed cultures were characterized by low viscosity, which presumably allows a better mass transfer and oxygen supply. Ishikawa et al. [88] used *Acinetobacter* ST-550 strain able to indigo production immobilized on a polyurethane carrier by a novel immobilization method with the use of the adhesive bacterionanofiber protein AtaA from the trimeric autotransporter adhesin (TAA) family. As obtained results showed the immobilized cells were able to faster indigo production rate at high concentration of substrate compared with their planktonic counterparts. 

Immobilization of whole bacterial cells has also been used to improve lactic acid fermentation by, e.g., *Lactobacillus helveticus*, *L. rhamnosus*, *L. delbrueckii* subsp. *bulgaricus*, *L.* *casei* and *Streptococcus salivarius* [13]. The most widely used methods for the immobilization of lactic bacteria are Ca-alginate and *k*-carrageenan entrapment. Zhao et al. [69] immobilized *Lactobacillus rhamnosus* on mesoporous silica-based material, which allowed them to overcome the destruction of the carrier by lactic acid. *L. rhamnosus* encapsulated in this manner showed high operational stability and lack of changes in lactic acid production yields in up to eight repeated batches [69]. Immobilization of lactobacilli on resin and silica supports may also result in a significant improvement of bacteriocins production [89].

Various techniques of cell immobilization are also useful in the production of enzymes such as α-amylase, β-amylase, xylanase, glucoamylase, pullulanase or alkaline phosphatase [13,90,91]. Viable, but non-growing immobilized *Escherichia coli* cells in biocatalytic films developed by Lyngberg et al. [53] produced β-galactosidase with higher specific activity compared to suspended cells. Similar observations were made by Klingeberg et al. [54]: α-amylases and pullulanases produced by gel entrapped bacteria were characterized by higher specific activities than those obtained from free cells.

### 3.3. Biodegradation/Biotransformation Capacity of IC Systems

The biodegradation/biotransformation of toxic pollutants and xenobiotics is one of the major applications of immobilized cell systems. Immobilized cells are characterized by better biodegradation efficiency than free cells. The poor capabilities of xenobiotics degradation by microorganisms in the suspended state are mainly attributed to the irreversible binding of pollutants by extracellular polymeric substances, which hinder the effective transport [92,93]. Moreover, free cells degrading pollutants by metabolic activity are not stationary and not adapted to survive under mechanical and environmental stress [1]. Immobilized cells systems are characterized by displayed higher volumetric and lower specific degradation properties than free suspended cells [11]. Techniques of whole cell immobilization in suitable carriers protected bacteria from shock load application and the toxic effects of xenobiotic compounds or their metabolites. In wastewaters the organic compounds first adsorb onto the surface of the supports and then gradually penetrate via its pores. This allows the microorganisms to release extracellular enzymes for the pre-hydrolysis of organic xenobiotics, and then to transport the fragments of pollutants through the cellular membrane for oxidation [93,94,95]. 

The majority of studies concerning the increased biodegradation capacity of immobilized cells refer to phenol and its chlorinated derivatives [11,94,96]. Presumably, the observed enhanced ability of degradation is mostly due to the reversible adsorption of the pollutant on the carriers, e.g., polyurethane or chitosan [45,97]. Adsorption of toxic compounds decreases the overall toxicity level, which normally results in inhibitory effect on free cells [10]. *Acinetobacter* sp. strain AQ5NOL 1 was immobilized by Ahmad et al. [98] in gellan gum, and as results showed, at phenol concentration of 100 mg/L, both free and immobilized bacteria exhibited similar capabilities of phenol degradation, whereas at higher phenol concentrations the immobilized cells were characterized by a higher rate of degradation. Chung et al. [56] observed that in the case of *Pseudomonas putida* CCRC14365 strain, due to the substrate inhibition effect, suspended cells were capable of phenol degradation only at a dose of 600 mg/L, whereas immobilized cells tolerated levels up to 1000 mg/L. Results of numerous studies have indicated that immobilization of bacterial cells may be a good alternative offering higher degradation ability at high doses of phenol. 

*Pseudomonas* strain encapsulated in a polyacrylamide gel matrix has also been used for uranium and Cu(II) uptake [99,100]. Immobilization also includes anaerobic species, e.g., *Thermus aquaticus* YT-1, a good producer of extracellular proteases [101], anaerobic sulfate reducing bacteria (SRB) [102], and annamox bacteria [83]. 

It has been documented that IC systems show greater catalytic stability and often tolerate higher concentrations of toxic compounds than suspended cells. This ability is attributed to the increased resistance of immobilized cells to toxic pollutants or other compounds, which results more from the protective effect of the supports than the modified physiological properties of immobilized cells. The encapsulation of bacteria protects them from various environmental stressors by the creation of a more stable microenvironment for the entrapped cells [15]. The protective effect of the beads matrix is also observed during drying and rewetting cycles. Diffusional properties and the restricted volume of beads that control the volume and rate of water reaching beads additionally decreases the chance of osmotic shock [10]. Encapsulation in beads also maintains the oxidative properties of some species in a pH- and temperature-independent way. It is noteworthy that carriers provide protection not only against variable environmental conditions, but also against predators, mainly due to physical restriction—the size of pores, ranging from 2 to 6 μm, prevents predator access [16].

Nevertheless, Diefenbach et al. [44] suggested that the increased resistance of immobilized cells to toxic compounds, antibiotics, biocides and other antimicrobial agents is mainly attributed to changes in membrane permeability, and the composition and architecture of the cell wall and membrane, e.g., incorporation of saturated fatty acids.

### 3.4. Nucleic Acids Content/Plasmid Stability

Another interesting modification observed in immobilized cells concerns changes in the total content of nucleic acids. Immobilization presumably stabilizes the protein synthesis capacity of microorganisms, which results in increased total RNA content. This assumption is confirmed by studies conducted by Lyngberg et al. [53]. Many genes encoding enzymes involved in the degradation of pollutants are carried on plasmids, and thus increased plasmid stability is a desirable feature in immobilized cells used for the bioremediation of soil or groundwater [103,104]. Chen et al. [62] noticed that effects of immobilization on bacterial physiology should be particularly considered in the case of genetically modified bacteria. One of the essential problems in obtaining high cell density and continuous high productivity of host mutant strains is plasmid stability. Barbotin [61] noticed that immobilization can increase the retention of plasmid-bearing cells and thus delay overgrowth by their plasmid-free counterparts. The stabilizing effect of immobilization with respect to nucleic acids has been observed for various carriers, e.g., polyacrylamide, polyvinyl alcohol, silica foam, glass and gelatin beads, agarose, Ca-alginate and *k*-carrageenan [10,62,105]. For immobilized *E. coli* cells cultured in non-selective media, factors affecting plasmid stability include the plasmid properties, host strains, growth rate, the number of plasmid copies, dilution rate and nutrient and oxygen limitations [61]. Nasri et al. [58] investigated three *E. coli* hosts for the pTG201 plasmid. Results showed increasing plasmid stability in immobilized cells compared to free counterparts, and what is important is that the authors excluded the notion that higher stability was due to the plasmid transfer between the immobilized cells. Sayadi et al. (1989) [59] observed higher plasmid stability in an immobilized *E. coli* recombinant, even in media deprived of glucose, nitrogen or phosphate. Some authors proposed oxygen diffusion limitations and compartmentalized growth of immobilized cells as factors explaining plasmid stability in IC systems [105,106]. These observations have been confirmed by several authors who investigated the influence of oxygen supply on plasmid stability, and in all studies stability was highest at 100% oxygen saturation [60,107]. Zaghlou et al. [108] investigated the stability of a multicopy plasmid that carried the *aprE* gene encoding alkaline protease. As results showed, plasmid stability reached 83% for *Bacillus subtilis* cells immobilized in alginate [108]. Recent reports on the higher retention of plasmid-bearing cells have further extended the scope of whole-cell immobilization to recombinant product formation.

## 4. Conclusions

The influence of cell immobilization on bacterial metabolism, and various applications of immobilized cells have been widely illustrated, although future studies should also focus on the engineering problems with immobilized cell system limitations, especially with difficulties in biomass transfer and effective diffusion. Bacterial cells in the sessile mode of growth have been characterized by their high capabilities of biodegradation and low susceptibility to antimicrobial agents and environmental stresses. The extraordinary properties of cells in the immobilized state, mainly increased metabolic activity, increased growth rate, plasmid stability and protection from toxicity, may contribute to wider microbiological applications of immobilization technology. Nevertheless, additional studies are necessary to identify the mechanisms responsible for the particular physiology of the immobilized cells.

## Figures and Tables

**Table 1:** Factors determining bacterial cells’ adsorption.

Support	Environmental Factors	Microbial Cell
Roughness	pH	EPS
Porosity	Oxygen concentration	Age of cells
Hydrophobicity	Temperature	Physiological state of cells
Superficial charge	Nutrient availability	Hydrophobicity
Toxicity	Flow velocity	Flagella, pilli
Type of functional groups	Cations/anions	Fimbriae, glycocalyx
	Antimicrobial agents	Surface proteins
	Hydrodynamic forces	
	Adhesive forces	
	Rheology	

**Table 2:** Components of the EPS and their role in biofilm formation.

Component of the EPS	Role	Typical Content	Ref.
Polysaccharides	Adhesion to abiotics/biotics surfaces, aggregation of bacteria, mechanical stability of biofilm, intracellular communication, retention of water, adsorption of organic and inorganic compounds, protective barrier to antibiotics, bacteriophages, predators, bacteriocins, ionic exchange, growth substrates	40%–95%	[2,22,29,30,31,32,33]
Proteins	Adhesion, aggregation of bacteria, enzymatic activity, retention of water, tolerance to dry, sorption of organic and inorganic compounds, electron and donor acceptor, extracellular electron transfer mediated by matrix-associated proteins, ionic exchange, protective barrier	up to 60%	[2,22,29,30,31,32,33,34,35,36]
Nucleic acids	Adhesion, exchange of genetic information, export of cell components, horizontal gene transfer, growth substrates	up to 10%	[22,29,30,31,32,33]
Lipids	Flocculation, biosorption	up to 10%	[2,29,30,31,37]
Humic substances	Electron donors or acceptors	up to 30%	[29,37]

**Table 3:** Metabolic responses of immobilized cells.

Metabolic Responses	Possible Explanation	Ref.
Increased growth rate	Nutrients adsorbed on surfaces	[4,8,10,11,12,13]
	Support protection	
	Detoxification of inhibitors	
	pH buffering by ion exchange	
Decreased growth rate	Mass transfer limitation	[8,40,41,42]
	Diffusion limitation	
	Oxygen/nutrients gradient	
	Lack of nutrients adsorbed on surfaces	
Increased adhesion of cells	Cell hydrophobicity	[4,21]
Higher productivity	Support protection	[1,10,11,12]
	Increased tolerance to inhibitors and toxic compounds	
Lower substrate affinity	Diffusion limitation	[4,43]
Altered pH	Differences between proton concentration at surface and in the bulk phase	[4,10]
Increased tolerance/resistance to inhibitors	Support protection	[10,16,33,44,45]
	Detoxification of antibacterial substance	
	Alterations in composition and organization of cell wall and cell membrane	
	Higher protein-to-lipid ratio in membranes	
	Modification of membrane porins	
	Heat shock proteins (HSPs) and biosurfactants production	
	Point mutations	
	Horizontal gene transfer of resistance genes	
Changes in protein production/different genes expression	Differences in types and ratio of proteins involved in biofilm formation, attachment of bacteria, amino acids and cofactors biosynthesis, adaption and protection of cells, variable genes expression within biofilms, planktonic and immobilized cells, increased invasiveness of immobilized cells	[1,11,12,46]

**Table 4:** Metabolic responses to immobilization in several bacterial species.

Bacterial Species	Immobilization Technique	Physiological Responses	Ref.
*Nitrobacter* sp.	Anion-exchange resin beads	Production of extracellular slime layer	[48]
*Escherichia coli*	Entrapment	Higher specific activity of enzyme; slower degradation of RNA	[53]
*Clostridium thermosaccharolyticum*	Entrapment in Ca-alginate	Higher specific activity and productivity of starch hydrolyzing enzymes	[54]
*Marinobacter* sp.	Porous glass beads	Increased metabolizing of c_18_-isoprenoid ketone; shorter generation times; higher CO_2_ production	[50]
*Listeria monocytogenes*	Gel Cassette System	Decreased growth rate	[4,41]
*Escherichia coli*	-	More oxidized glucose metabolites	
*Streptomyces violatus*	Sponge-cubes	Higher antibiotic production	[55]
Lactic acid bacteria	Ca-alginate, *k*-carrageenan beads	Increased lactic acid production	[13]
*Acinetobacter* sp. *Pseudomonas putida*	Gellan gum, chitosan, polyurethane	Phenol and chlorophenol biodegradation	[56]
*Methanosarcina burkeri*	Ca-alginate	Increased methane reduction rate	[57]
*Escherichia coli*	Polyacrylamide, polyvinyl alcohol, silica foam, glass and gelatin beads, agarose, Ca-alginate, *k*-carrageenan	Enhanced plasmid stability	[58,59,60,61,62]
